# Clinical Translation of Cell Therapies in Stroke (CT2S) Checklist—a pragmatic tool to accelerate development of cell therapy products

**DOI:** 10.1186/s13287-021-02147-6

**Published:** 2021-01-29

**Authors:** Anjali Nagpal, Austin G. Milton, Simon A. Koblar, M. Anne Hamilton-Bruce

**Affiliations:** 1 Stroke Research Programme, Adelaide Medical School, University of Adelaide, Adelaide, South Australia 5005 Australia; 2 Stroke Research Programme, The Queen Elizabeth Hospital, Woodville South, South Australia 5011 Australia; 3 Royal Adelaide Hospital, Central Adelaide Local Health Network (CALHN), Adelaide, South Australia 5000 Australia; 4 Stroke Research Programme, Neurology, Central Adelaide Local Health Network, Adelaide, South Australia Australia; 5 Stroke Research Programme, Neurology 5C, The Queen Elizabeth Hospital, Central Adelaide Local Health Network, Woodville South, South Australia 5011 Australia

**Keywords:** Clinical translation, Cell therapies in stroke, Checklist, Study design, Regulatory, Patient and Public Involvement in Research (PPIR), Health economic

## Abstract

**Background:**

Cell therapies present an exciting potential but there is a long history of expensive translational failures in stroke research. Researchers engaged in cell therapy research would benefit from a practical framework that can help in planning research and development of investigational cell therapies into viable medical products.

**Methods:**

We developed a checklist using a mixed methodology approach to evaluate the impact of study design, regulatory policy, ethical, and health economic considerations for efficient implementation of early phase cell therapy studies.

**Results:**

The checklist comprises a series of questions arranged under four domains: the first concerns study design such as characterization of target study population, trial design, endpoints and operational fit of dosage, time, and route of administration to target populations. A second domain addresses the data package required for regulatory approval relevant to the intended use (allogeneic/autologous; homologous/non-homologous; nature of cell processing). The third domain comprises patient involvement to ensure relevant data is collected via targeted study design. The final domain requires the team to determine the critical data elements that could be built into study design to enable health economic data collection to be started at an early phase of the study.

**Conclusions:**

The *CT2S* checklist can help to determine areas of expertise gaps and enable research groups to appropriately allocate resources for capacity building. Use of this checklist will allow identification of key areas where trial planning needs to be optimized, as well as helping to identify resources that need to be secured. The *CT2S* checklist can also serve as a general cell therapy research decision aid to improve research output and accelerate new cell therapy development.

**Supplementary Information:**

The online version contains supplementary material available at 10.1186/s13287-021-02147-6.

## Background

The landmark success in the clinical translation of reperfusion strategies in stroke, thrombolysis and endovascular thrombectomy, represents significant mortality and morbidity benefit [1]. However, these therapies are limited by a narrow window of opportunity [1, 2]. Despite declining mortality and morbidity due to general improvements in systems of stroke care [3], there is a present unmet need for impactful new therapeutic strategies in addition to rehabilitation to result in meaningful decrease in long-term disability [4]. Stroke research has yielded numerous promising preclinical therapeutic candidates as the understanding of brain injury and recovery following stroke at molecular, cellular, and systems levels evolved [5–11]. Early phase studies of cell therapies (CTs) in human stroke subjects indicate functional benefit and credible safety to date [11]. There is a scientific imperative for a successful clinical translation of cell therapy into relevant treatment strategies for patients with stroke-related disability [12]. Stem cell Therapies as an Emerging Paradigm in Stroke (STEPS) recommendations proposed high-level consensus-based pragmatic concepts underpinning the development of CTs in stroke [13]. Feedback sought from researchers and industry highlighted that the lack of awareness of clinical development pathways may potentially lead to the loss of many innovative developments in this field [14]. Thus, groups engaged in cell therapy research would benefit from an expanded and practical framework that can help accelerate research and development of investigational CTs into a viable medical product.

## Methods

In our earlier research which underlies our work here, a mixed methodology approach was used to explore and evaluate the impact of study design, regulatory policy, ethical, and health economic considerations for efficient implementation of early phase cell therapy studies. Here, we critically appraised the outcomes of the afore-mentioned research or relevant quality determinants of early phase clinical studies investigating cell therapy in stroke [15]. They were then integrated into a practical framework in checklist form organized under four domains: study design, regulatory, ethical, and health economic considerations, proposed as a tool for research teams to efficiently plan operationalization of early clinical studies of cell therapy. A brief summary of the methods used in each of the four domains follows here.

### Study design

A systematic review of published early phase clinical studies with CTs in ischemic stroke was undertaken to understand the choice of study design elements, endpoints, cell therapy characteristics, dose, and mode of delivery and to analyze their impact on the quality of evidence generated [11]. Meta-analysis of this data was performed by a statistician using STATA/SE v14.1 (StataCorp LP, College Station, TX, USA). The protocol for the review is detailed in PROSPERO: International prospective register of systematic reviews [16]. An additional question on medication has been included in response to peer review.

### Regulatory policy

Regulatory provisions that aim to ensure adequate oversight of the development of regenerative medicine products were sought [17]. The following databases were searched to identify relevant studies: PubMed/MEDLINE, Google Scholar, Scopus, Web of Science, Cumulative Index to Nursing and Allied Health Literature (CINAHL), Cochrane, and websites of regulatory bodies in various countries and legal literature databases such as AustLII and Thomson Reuters Westlaw. Reference lists of all identified reports and articles were hand-searched for additional studies. Keywords included ischemic stroke, stem cells, cell therapies, regulatory policy, public policy, and regenerative medicine. National regulations from different countries were reviewed and analyzed for key themes. These themes indicate considerations that are critical to successfully navigate the requirements of the regulatory pathways across the globe.

### Ethical considerations

A qualitative interview-based study was undertaken [18] with stroke survivors to collect insights and understand ethical perspectives on the research design of our proposed early phase cell therapy study in chronic ischemic stroke [19]. This was conducted via semi-structured, face-to-face interviews on specific aspects of the design of the study while a concurrent thematic analysis was conducted to identify key perspectives until data saturation was achieved. Issues included the relevance and attitudes towards cell therapy research in chronic ischemic stroke, consent issues with participation in such research, and the relevance of planned outcome measures to individuals who have had personal experience of ischemic stroke.

### Health economic considerations

To assess the breadth of economic evaluation undertaken on the use of CTs in stroke, a systematic review was conducted to appraise the quality of evidence generated by these studies [20]. Because there was minimal data on stroke alone, the scope of our review was broadened to include neurological disorders. The review examined studies published in English between 2007 and 2017 that performed an economic evaluation of the use of stem cells in adult patients with neurological diseases. Data analyzed and reported included study population, disease indication, main analytical approaches for the economic analysis and perspective, key assumptions made or tested in sensitivity analyses, cost outcomes, estimates of incremental cost-effectiveness, and approaches to quantifying decision uncertainty. The protocol for this was defined a priori in PROSPERO: International prospective register of systematic reviews [21]. Key emergent data considerations were thereafter included in the proposed checklist.

## Results

Our *CT2S* checklist, summarized in Table 1, can serve as a tool for research teams to plan efficient operationalization of early clinical studies in stroke (the full checklist, complete with questions, is provided in Supplement 1).

**Table 1:** Clinical Translation of Cell Therapies in Stroke (CT2S): Checklist for efficiency in trial conduct

Domain	Topic	Number of questions
**Study design**	Target population	4
	Trial design	6
	Study endpoint	3
	Timing of study after stroke	4
	Intervention	4
	Medication	1
	Comparator	3
	Statistical analysis	2
	Safety reporting	1
**Regulatory**	Intended cell therapy use	4
	Cell therapy processing	9
	Expertise in submission of clinical trial applications to regulators	1
**Ethical**	Patient and Public Involvement in Research (PPIR)	10
	Study safety committee	1
**Health economic**	Cost outcomes	5

### Study design considerations

Randomized clinical trials (RCTs) determine average treatment effects in a target population and rely on the deliberate selection of a homogenous population to study and compare, to reduce the effect of bias, confounding and effect modification [22]. However, the suitability of RCTs has often been challenged in areas of clinical research such as stroke wherein the effect of a stroke injury is a sum of individualized patterns of injury that is influenced by numerous patient and management factors [23, 24]. In this context, the application of CTs that inherently rely on the crosstalk in an injured brain environment to facilitate recovery poses challenges when analyzing global functional outcomes; it also represents a challenge to recruit a homogenous patient population in the large numbers needed to ensure sufficient power [25]. This is compounded by the fact that the rate and degree of spontaneous recovery expected differs between individuals based on initial severity, initial neuronal reserve (influenced by factors such as co-morbid medical conditions and genetic factors), timing, and appropriateness of other established stroke interventions [25]. It is therefore plausible to consider that the overall stroke population is a composite of smaller sub-groups defined by shared characteristics such as the dominant functional system impaired or predicted trajectory of recovery [26]. STEPS III recommended that evaluation of stem cells would benefit from the use of domain-specific end points, i.e., analysis of change in relevant domains of functioning would likely result in more clinically relevant data [13]. Adoption of pragmatic research methodologies may generate clinically relevant data to support more targeted development in this field [25–29], such as the following:

#### Adaptive trial enrichment designs

Use of randomized trial designs that adaptively change enrolment criteria during a trial, adaptive enrichment designs can potentially provide clinically relevant information about which subpopulations are likely to benefit from CTs [26–28]. These subpopulations could be identified utilizing baseline functional or prognostic phenotypes or structural, functional, or imaging biomarkers [26]. The trial population could be enriched to a greater proportion of subjects with a potential for benefit from the treatment [28]. Furthermore, this allows for the early stopping of trial exposure in subpopulations that do not demonstrate any benefit—an aspect especially relevant to CTs [27]. In the context of early phase cell therapy research, this approach can enable researchers to have increased power to detect and measure true effect size in the subpopulations that show benefit and improve efficiencies in designing subsequent confirmatory trials [27, 28]. The recent DEFUSE 3 study used this methodology to evaluate endovascular treatment in ischemic stroke patients with imaging-perfusion mismatch and provides useful insights for future studies investigating CTs [29].

#### *C*luster randomization

Cluster randomization has been used in stroke rehabilitation trials to evaluate the treatment impact of interventions in different patient groups clustered by site or patient characteristics [30]. The key premise here is that clusters share common characteristics and that observations on individuals in the same cluster tend to be correlated [31]. This variance can be accounted for in study planning by incorporating the design effect into sample size calculations. Design effect depends on the average cluster size and the degree of correlation within clusters (intracluster correlation coefficient or ICC). ICCs for outcome measures such as the Barthel Index (BI), modified Rankin Score (mRS), and National Institutes of Health Stroke Scale (NIHSS) have been calculated previously and can be used in planning future studies that use these models [32]. Clinical guidelines recommend the delivery of rehabilitative interventions individualized to specific patient needs as a key component of stroke management [33]. There is evidence that CTs and rehabilitation may have a mutually facilitative biological effect [34]. Rehabilitation has been recommended as a necessary concomitant therapy in clinical studies investigating CTs [13]. In the context of designing future effectiveness studies with CTs, it may be useful to consider cell therapy in combination with rehabilitation as an integrated therapeutic package. While this may have labeling implications, it makes clinical sense intuitively. Randomizing patients into clusters with similar rehabilitative needs, based on similar functional deficits, may enable delivery of rehabilitation that is targeted, yet can be standardized enough to enable robust evaluation of the effect of cell therapy.

#### The choice of outcome measures

The majority of cell therapy studies to date enrolled patients with middle cerebral artery infarction that results in predominantly sensory-motor deficits [11]. It is interesting to note that only six of the 26 studies utilized outcome measures that specifically measured change in motor impairment [11]. In most studies, the change in disability following CTs administration was evaluated using either a global measure of impairment such as NIHSS, or a measure of activity dependency such as mRS or BI [11]. While these studies followed conventional advice from stroke trials to use established (hard) global disability endpoints, future effectiveness studies may be more efficient if the primary outcome measure chosen is domain-specific and aligned to specific impairment in the selected study population [35]. This endpoint could be supported by secondary endpoints such as changes in domains of activity and participation. Regulatory agencies have communicated their willingness to accept such choices in effectiveness studies as these would hopefully provide clearer data guidance for eventual clinical use [36].

### Regulatory considerations

Regulatory agencies across the globe are cognizant of the paradigm change in medicine that CTs potentially represent in terms of being able to generate replacements for cells that are lost to injury or disease [36]. CTs are technically challenging to develop, when compared to conventional pharmaceutical products. Their clinical use is likely to be more akin to cell and organ transplant products, making their development fit better with academic institutes than conventional manufacturers [17].

Academic centers, with their scientific expertise in diseases with high unmet need and relatively easy access to patient cohorts and their clinical samples and imaging data, are well placed to lead research in innovative areas like CTs [37]. Driven by scientific principles, they are often limited in their capability to analyze an investigational product in terms of its “target product profile” and to implement required standardization and quality control processes essential to product quality assurance requirements for Good Manufacturing Practice (GMP) and Investigational Medicinal Product Dossier compliance [17, 37]. These aspects need to be established in early phases of development as it may be almost impossible to develop these at a later stage without having to repeat the whole journey. However, most research institutions have sparse resources available to enable access to regulatory expertise to facilitate appropriate clinical trial planning and regulatory approval process, as the pathways for assessment and oversight differ substantially, depending upon the exact conditions of clinical use [17, 37].

### Ethical considerations

Patient and Public Involvement in Research (PPIR) is an emerging field that aims to evaluate whether research investment represents value from a patient’s (the ultimate beneficiary) perspective [38]. Studies have reported a general positive attitude towards participation in cell therapy research, although with a wide divergence in efficacy expectations between study participants and research teams [39]. Our study revealed that patients had a pragmatic realistic approach to anticipated benefits [18]. It was interesting to note that patients were more worried about a loss of function as a possible risk as compared to more widely discussed risks such as tumorigenicity and death [18]. The importance of measuring changes in often neglected outcomes such as cognition, mood, and overall ability to restore “normal” pre-stroke participation levels is a key insight that should inform future trial designs [18].

### Health economic considerations

Lack of availability of cost outcomes data with CTs may make demonstration of value proposition for these innovative but high-cost therapies extremely challenging. Lack of access may represent the last component of the widely acknowledged “translation roadblock” for CTs even after the demonstration of clinical benefit [40].

A systematic review reported that only three studies have been published on the cost-effectiveness analysis of use of cell therapy [20]. All three studies conducted a cost-utility analysis using decision-analytic models and reported an incremental cost per quality-adjusted life-years gained. A modeling study in stroke using expert opinion on probable effect size of cell therapy reported overall societal value driven by long-term cost savings due to decreased cost of disability and productivity losses [20]. Incorporation of health economic considerations in early stages of research with CTs can serve two purposes [40]:

1. To inform researchers and developers about the regulatory and reimbursement strategy, using early-stage (or iterative) health economic modeling including headroom analysis, and
2. To estimate unknown effect sizes and beliefs using methods like stakeholder preference elicitation and multi-criteria decision analysis to refine trial designs and maximize the generation of acceptable cost.

## Discussion

Stroke represents one of the leading causes of disease burden in Australia and across the globe [4]. CTs represent an exciting option with the demonstration of neurovascular repair and abrogation of neuroinflammation in preclinical stroke models and exploratory clinical studies [5, 10]. Despite a robust amount of exploratory clinical trial data published over the last decade, only a minimal percentage of these cell therapies have progressed further towards medical product development [41]. Given a long history of expensive translational failures in stroke research and the fact that CTs research has been driven by academic research groups primarily, there is an urgent need to identify and address key factors that can enable efficient execution of clinical development of cell therapy products in stroke [37].

The *CT2S* checklist is a decision aid for all research groups engaged in cell therapy research to improve their research output and enable them to lead cell therapy product development effectively in stroke. The list describes different aspects critical to the development of innovative cell therapies and that need to be addressed by development teams in the course of planning for early phase clinical studies. It is important to note that while the checklist is primarily targeted for use in academic institutions, these determinants are equally critical in the industry setting, as the need for the proposed framework is likely to be higher in these settings.

It would be useful to validate the utility of this checklist with key stakeholders such as academic researchers, clinicians, patient advocacy groups, patients, ethics committees, regulators, and Health Technology Assessment (HTA) experts. The major part of evidence in the early stages of clinical translation of CTs is derived from small-sized studies. Database resources such as the Virtual International Stroke Trials Archive (VISTA) database, established to serve as a repository of anonymized data from completed clinical trials in stroke, are already functional [42]. Creating a database for cell therapy trials may be a way forward to collate data across numerous studies that could potentially advance our understanding of the efficacy and safety profile of CTs. In this context, the *CT2S* checklist can also serve to enable standardization of data collection, which in turn may optimize the value of these cell therapy trial databases.

The *CT2S* checklist challenges conventional clinical trial design approaches and relates the challenge of CTs in stroke recovery trials and determinants of execution to the broader framework of policy advancement, economic advantages, and trial design. It is important to note that some components of this checklist are relevant beyond stroke research to critical factors affecting wider cell therapy research. Thus this checklist can be adapted to the requirements of other disease areas, thereby providing a tool to accelerate all cell therapy research.

## Conclusions

The *CT2S* checklist represents a practical framework that can help research teams to identify existing capacity gaps and build financial and human resources to improve their cell therapy translational programs. This will potentially accelerate clinical translation of these innovative therapies that hold a promise, if successful, of ushering a paradigm change in stroke treatment.

## Supplementary Information

**Additional file 1.** Clinical Translation of Cell Therapies in Stroke (CT2S): Checklist for efficiency in trial conduct.

## Data Availability

The complete CT2S Checklist generated by this method is available as a Supplementary Information file available at 10.1186/s13287-021-02147-6.

## Abbreviations

BI: Barthel Index
CT2S: Clinical Translation of Cell Therapies in Stroke
CTs: Cell therapies
DEFUSE: Diffusion and Perfusion Imaging Evaluation for Understanding Stroke Evolution
GMP: Good Manufacturing Practice
HTA: Health Technology Assessment
ICC: Intracluster correlation coefficient
mRS: Modified Rankin Scale
NIHSS: National Institutes of Health Stroke Scale
PPIR: Patient and Public Involvement in Research
RCTs: Randomized clinical trials
STEPS: Stem cell Therapies as an Emerging Paradigm in Stroke
VISTA: Virtual International Stroke Trials Archive
