# Supplementary material for: Clinical Translation of Cell Therapies in Stroke (CT2S) Checklist—a pragmatic tool to accelerate development of cell therapy products
Source: Stem Cell Res Ther. 2021 Jan 29;12:93. doi: 10.1186/s13287-021-02147-6 (PMC7844985; doi:10.1186/s13287-021-02147-6)
Supplement: Supplementary file 1 — Additional file 1. Clinical Translation of Cell Therapies in Stroke (CT2S): Checklist for efficiency in trial conduct. [file 13287_2021_2147_MOESM1_ESM.docx]

| **Clinical Translation of Cell Therapies in Stroke (CT2S): Checklist for efficiency in trial conduct** | | | |
| --- | --- | --- | --- |
| **Domain** | **Topic** | **Item** | **Response** |
| Study | Target population | Is the intended patient population clearly identified (from preclinical and other exploratory studies)? |  |
| design |  | Is the applicable patient population yet to be adaptively selected in the trial due to the potential patient and/or disease heterogeneity? |  |
|  |  | Are baseline characteristics of participants mapped to ensure alignment to primary impairment of interest, before randomization? |  |
|  |  | Is imaging required to support clinical examination for baseline assessment? |  |
|  | Trial design | Is the trial use group randomization by cohorts defined by impairment rather than lesion territory (clinical/imaging)? |  |
|  |  | Is there value in adopting adaptive trial design versus fixed RCT? |  |
|  |  | Does the trial design allow interim selections of design aspects (dose levels or sample size) or design parameters (effect threshold)? |  |
|  |  | Is the interim analysis pre-specified in trial protocol? |  |
|  |  | Are rules for efficacy/futility pre-specified in trial protocol? |  |
|  |  | Is the minimal clinically important difference for selected primary outcome measure established *a priori* and used to decide on efficacy/futility? |  |
|  | Study endpoint | Is the primary outcome measure appropriate to the primary impairment of interest? |  |
|  |  | Do secondary endpoints include domain specific outcome measures for activity & participation? |  |
|  |  | PROM incorporated as secondary endpoints? |  |
|  | Timing of study after stroke | Which is the preferred phase of stroke for use of the candidate cell therapy product? |  |
|  |  | What additional logistics challenges does this phase pose for cell therapy delivery? |  |
|  |  | Is cell therapy processing feasible for time of delivery after stroke? |  |
|  |  | Is the time window for delivery defined *a priori*? |  |
|  | Intervention | Is the cell therapy (dose/route) selected based on extrapolation from preclinical research or deduced using adaptive design? |  |
|  |  | Is rehabilitation intervention targeted to primary impairment of interest? |  |
|  |  | Is the dose of rehabilitation defined *a priori* in protocol? |  |
|  |  | Is temporal sequencing of cell therapy & rehabilitation used in study protocol? – justification from available evidence? |  |
|  | Medication * | Are concomitant medications that patients are on, being recorded?’ |  |
|  | Comparator | Is the control arm receiving placebo delivery? |  |
|  |  | Is the control arm undergoing sham procedure? |  |
|  |  | Is the intervention and dose of rehabilitation in the control arm matched to the experimental arm? |  |
|  | Statistical analysis | Does the statistical plan support adaptive design, if chosen, and pre-specify the interim analysis and study progress rules? |  |
|  |  | Does the statistical plan justify the analytical methods used for selected endpoint analysis? |  |
|  | Safety reporting | Is there a mechanism for long-term safety follow-up through extension studies/registries? |  |
| Regulatory | Intended cell therapy use | Is it allogeneic or autologous? |  |
|  |  | Is it homologous/non-homologous? |  |
|  |  | Is cell therapy combined with tissue engineering products or devices? |  |
|  |  | Would cell therapy delivery need invasive procedure and/or use of delivery devices? |  |
|  | Cell therapy processing | Is the cell therapy manipulation 'minimal'? |  |
|  |  | Will cell therapy be processed on-site or sourced from cell processing facility/sponsor manufacturing site? |  |
|  |  | Does the site/outsourced facility have required manufacturing approvals? |  |
|  |  | Does the site/outsourced facility have GMP-compliant cell-processing protocols? |  |
|  |  | Does the site/outsourced facility have cell therapy specific release criteria? |  |
|  |  | Do the release criteria include structural and functional characterisation using validated assays? |  |
|  |  | Do the release criteria include infection screening? |  |
|  |  | Does the site/outsourced facility have required quality control processes in place? |  |
|  |  | Does the site/outsourced facility have required cryopreservation protocols to store and transport cell therapy? |  |
|  | Expertise in submission of clinical trial applications to regulators | Does the team have access to expert support for regulatory submissions and negotiations? |  |
| Ethical | Patient and Public Involvement in Research (PPIR) | Has any PPIR been conducted/planned for the proposed cell therapy study? |  |
|  |  | If yes, what aspects of study design were evaluated in PPIR? |  |
|  |  | Does the study protocol address the findings from the PPIR? |  |
|  |  | Is there patient/public representation on the study steering committee? |  |
|  |  | Were patient materials such as information sheet and other recruitment materials reviewed with patient/public representatives? |  |
|  |  | Are endpoints measuring change in participation, independence of living, mood, pain and fatigue included in study protocol? |  |
|  |  | Has a lead-in period to optimise secondary prevention in participants prior to participant randomisation, been considered in the study protocol? |  |
|  |  | Is formal cognitive assessment performed for all potential participants prior to consent? |  |
|  |  | If cognitively impaired patients are included in study, is the process for proxy consent defined in the protocol and approved by relevant ethics committees? |  |
|  |  | If implementing an adaptive research design, has the study team considered a dissemination strategy for changes in study conduct following prespecified interim analysis? |  |
|  | Study safety committee | Does the study committee include patient representation? |  |
| Health | Cost outcomes | Is resource use data for study and comparator groups defined in the protocol? |  |
| economic |  | Is data on direct costs of intervention/comparator defined and collected in the protocol? |  |
|  |  | Is data on direct costs of concomitant medications/interventions defined and collected in protocol? |  |
|  |  | What indirect costs data can be collected during the study period, and during study follow-up? |  |
|  |  | Is a PROM instrument that measures work productivity included in the study? Justification for inclusion/exclusion of PROM? |  |

* Included following peer review, as medications taken by patients can affect the outcome of cell therapy.

Abbreviations:

GMP Good Manufacturing Practice

PPIR Patient and Public Involvement in Research

PROM Patient-Reported Outcome Measures

RCT Randomised Clinical Trial
